# Artificial intelligence propels lung cancer screening: innovations and the challenges of explainability and reproducibility

**DOI:** 10.1038/s41392-024-02111-9

**Published:** 2025-01-24

**Authors:** Mario Mascalchi, Chiara Marzi, Stefano Diciotti

**Affiliations:** 1 Experimental and Clinical Biomedical Sciences “Mario Serio”, University of Florence, Firenze, Italy; 2 Department of Statistics, Computer Science, Applications “G. Parenti”, University of Firenze, Firenze, Italy; 3 Department of Electrical, Electronic, and Information Engineering “Guglielmo Marconi” - DEI, University of Bologna, Cesena, Italy; 4 Alma Mater Research Institute for Human-Centered Artificial Intelligence, University of Bologna, Bologna, Italy

**Keywords:** Lung cancer, Cancer imaging

In a recent study published in *Nature Medicine*, Wang, Shao, and colleagues successfully addressed two critical issues of lung cancer (LC) screening with low-dose computed tomography (LDCT) whose widespread implementation, despite its capacity to decrease LC mortality, remains challenging: (1) the difficulty in accurately distinguishing malignant nodules from the far more common benign nodules detected on LDCT, and (2) the insufficient coverage of LC screening in resource-limited areas.^1^

**Fig. 1:**
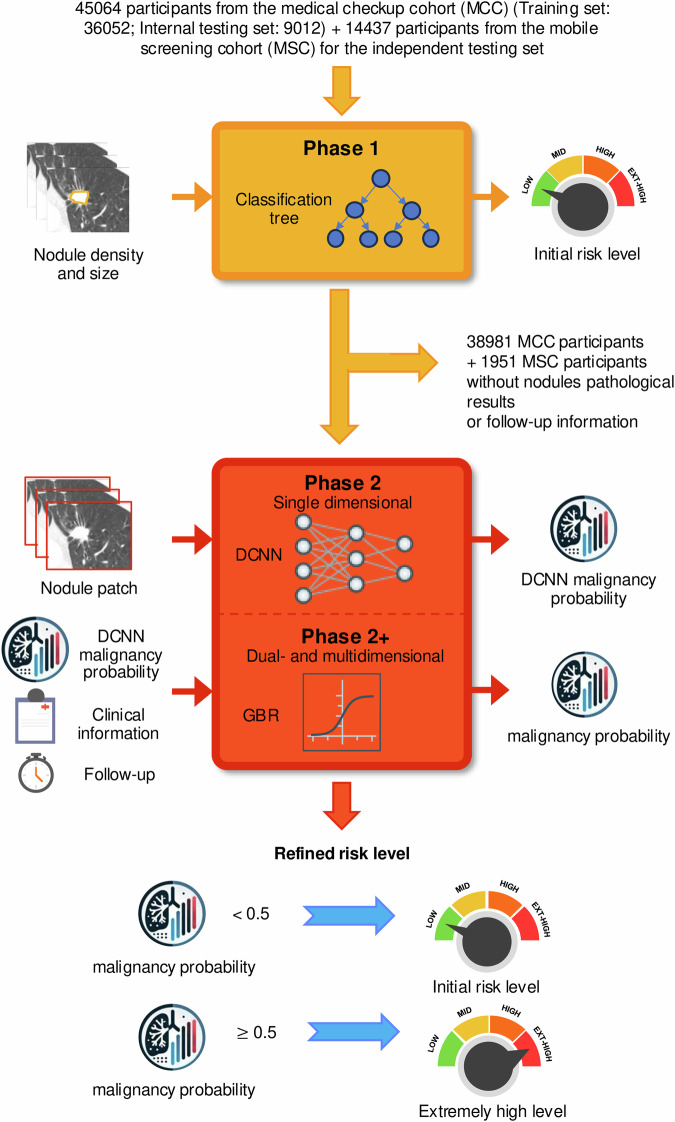
Illustrative workflow of the dual-phase malignancy prediction model. In Phase 1, a classification tree utilizes nodule density and size to provide an initial risk classification. Phase 2 refines these predictions using a DCNN-based model, which generates a malignancy probability from a 3D CT nodule patch. Finally, Phase 2+ incorporates additional clinical and follow-up features through a gradient boosting regression model, categorizing risk levels into low, mid, high, or extremely high based on predefined thresholds. (DCNN deep convolutional neural network; GBR gradient boosting regression; ext-high extremely high)

To perform nodule risk stratification, Wang et al. developed and validated a multi-step, multidimensional artificial intelligence (AI)-based system (Fig. 1) and introduced a data-driven Chinese Lung Nodules Reporting and Data System (C-Lung-RADS).^1^ A Lung-RADS system was developed in the US to stratify lung nodules into categories of increasing risk of LC and to provide corresponding management recommendations. This approach limits the need for further investigations to a minority of nodules, thereby reducing anxiety and minimizing the potential harms associated with uncertain LDCT findings for most individuals. The introduction of the C-Lung-RADS is justified by the unique characteristics of the Chinese screening population, which includes younger individuals, often without a smoking history—distinct from the predominantly older smoking-related cohorts in Western countries. The system’s phase 1 mainly focuses on identifying individuals with low-risk nodules using easily accessible features such as nodule size and density, and Phases 2 and 2+ are dedicated to better stratifying individuals with mid to extremely high-risk nodules. Specifically, Phase 2 (single-dimensional) integrates an image-based malignancy probability score using a deep convolutional neural network (DCNN) model, and Phase 2+ adds demographic and clinical data through a gradient-boosting regression model (Phase 2+/dual-dimensional) and incorporates nodule specific growth rate and volume doubling time from serial LDCT scans (Phase 2+/multidimensional). The complete AI pipeline demonstrated high performance, achieving an area under the receiver operator curve (AUC) of 0.918 and a sensitivity of 85.1% in the internal testing set and an AUC of 0.927 and a sensitivity of 85.6% in the independent testing set. Importantly, the AI-based system was integrated with mobile CT units to expand LC screening coverage in resource-limited areas.^1^

However, the performance of the AI-based system on the independent test set warrants further consideration. While the transition from Phase 2/single-dimensional to Phase 2+/multidimensional shows appreciable performance gains on the internal test set, the same improvement is not evident on the independent test set. This raises questions about the added value of incorporating additional information, given the lack of a clear benefit. Indeed, while the sensitivity of the multidimensional system at the selected operating point on the ROC curve is significantly higher than that of the single-dimensional approach, the AUC was not, and the ROC curves were essentially indistinguishable. This could be likely because the system’s score calibration was performed on the training set rather than on an unseen validation set, which may have resulted in potential overfitting. Still, the study does not provide information on how performance varies between prevalent and incident nodules, despite evidence suggesting that predictive models perform significantly better for prevalent nodules.^2^ Additionally, the level of expertise of the radiologists used as a benchmark to evaluate the pipeline’s performance is not disclosed.

Moreover, in evaluating complex AI systems, explainability plays a crucial role beyond performance. While attention maps provide some level of explainability, their utility remains limited. In the sole figure presented, the class activation maps appear as an extended version of the nodule segmentation input to the DCNN, offering minimal interpretive value. Specifically, these maps fail to highlight whether particular regions—such as the solid component of a part-solid nodule—were particularly influential in the DCNN model’s decision-making process. Enhancing the interpretability of such models could further boost their utility and adoption in clinical practice.

Finally, for AI systems tackling complex challenges, like the one described by Wang et al.^1^, clear and detailed documentation—encompassing all essential aspects for understanding and reproducibility—is fundamental. Unfortunately, some critical steps in the study’s description remain incomplete. For instance, the description of the internal test set used to stop the loss function of the Phase 2 DCNN model lacks clarity, raising concerns about potential data leakage—one of the primary pitfalls in machine learning algorithms that can result in falsely inflated generalization performance metrics.^3^ While the authors have shared some code, it lacks key components, exact dependencies, and the trained DCNN model. This limits the ability to reproduce the results or to apply the final model to new cases. While restrictions on data access for legal reasons are understandable, a more comprehensive release of the source code, documentation, and DCNN model would have significantly enhanced the reproducibility and broader applicability of the system.

The article focused on an AI-based system for evaluating the malignancy risk of pulmonary nodules. However, LDCT and AI also enable the assessment of additional radiological features associated with common comorbidities, significantly enhancing the screening test’s capacity to deliver valuable health information. Among these, pulmonary emphysema and vascular changes—particularly coronary artery calcifications (CAC)—are paramount. Emphysema is a quantifiable variable that predicts LC risk and also serves as a strong predictor of overall and respiratory mortality in LC screening populations.^4^ Moreover, deep learning algorithms have been applied to assess CAC and epicardial fat to efficiently identify individuals at risk for cardiovascular disease (CVD) events and mortality.^5^ This is particularly relevant as CVD is a leading cause of death in subjects undergoing LC screening and appears especially attractive for the relatively young (mean age 47 years) population in the study’s training and internal testing datasets. In fact, these younger individuals could greatly benefit from the timely identification of increased CVD risk, enabling the adoption of primary and secondary prevention.

In conclusion, Wang et al. present an innovative AI-driven approach that enhances nodule risk stratification in the Chinese population and, when combined with mobile CT units, has the potential to expand LC screening coverage. However, addressing challenges related to the explainability and reproducibility of the AI approach is essential to maximize its clinical utility and impact. Integration with AI systems capable of evaluating comorbidities is also advocated.
